# Genome-wide identification and development of SSR molecular markers for genetic diversity studies in *Ilex asprella*

**DOI:** 10.3389/fpls.2025.1582154

**Published:** 2025-05-23

**Authors:** Jingchun Li, Changqian Quan, Rongchang Wei, Fan Wei, Qing Ma, Yuquan Huang, Meihua Xu, Danfeng Tang

**Affiliations:** ^1^ Guangxi Key Laboratory of Medicinal Resources Protection and Genetic Improvement/Guangxi Engineering Research Center of Traditional Chinese Medicine (TCM) Resource Intelligent Creation, National Center for Traditional Chinese Medicine (TCM) Inheritance and Innovation, Guangxi Botanical Garden of Medicinal Plants, Nanning, China; ^2^ School of Traditional Chinese Medicine, China Pharmaceutical University, Nanjing, China; ^3^ Cash Crops Research Institute, Guangxi Academy of Agricultural Sciences, Nanning, China; ^4^ Research and Development Center, China Resources Sanjiu Medical and Pharmaceutical Co., Ltd., Shenzhen, China

**Keywords:** *Ilex asprella*, SSR identification and development, germplasm resource, species identification, genetic diversity

## Abstract

**Introduction:**

*Ilex asprella* is a common Chinese herb widely distributed in South China with high medicinal value, and its genetic diversity assessment is a prerequisite for the utilization of germplasm resources.

**Methods:**

Based on the published genome of *I. asprella*, this study conducted genome-wide SSR identification and development and performed genetic diversity analysis on 25 germplasm accessions.

**Results and discussion:**

The results showed that a total of 137,443 SSR loci were detected across the whole genome of *I. asprella*. Six types of SSRs were obtained, and the dinucleotide and trinucleotide repeats were dominant, with dinucleotide repeat motifs accounting for 84.20% of the total markers and trinucleotide repeat motifs accounting for 12.22%. A total of 15 highly polymorphic primers were ultimately selected, including 13 dinucleotide primers and 2 trinucleotide primers. The allele distribution of SSR loci in the genome of *Ilex asprella* was uneven, and the heterozygosity of different loci varied; the fixation index (F) were all greater than 0, indicating that there was an excess of pure heterozygotes in this population; the genetic differentiation coefficient (Fst) was 0.192, and there existed a large amount of genetic differentiation; the mean value of gene flow (Nm) between different loci was 1.175, and there existed a certain degree of gene exchange in the population; the molecular analysis of variance (AMOVA) indicated that the variation of individuals was the main source of total variation. Genetic analysis revealed that the 25 samples can be divided into three populations. pop2 had the highest genetic diversity, followed by pop3, and pop1 had the lowest genetic diversity, suggesting that there were differences in the level of genetic diversity among the populations. Overall, we found that there was a large genetic differentiation in the *Ilex asprella* population, a high level of genetic diversity, gene exchange between different populations, and high inter-population gene mobility, which was of guiding significance for the subsequent selection and breeding of new varieties of *Ilex asprella*.

## 1 Introduction

*Ilex asprella* (Hook. et Arn.) Champ. ex Benth, commonly known as Gangmei in Chinese, belongs to the holly genus and is primarily cultivated in Guangdong, Guangxi, Fujian, Jiangxi, and other regions (Wei et al., 2023). Gangmei was first recorded in “Sheng Cao Yao Xing Bei Yao”, authored by He Jian during the Qing Dynasty. Initially, only its roots were used medicinally. However, due to the scarcity of medicinal resources and the expansion of clinical applications, its stems have also been incorporated into medicinal use. The chemical constituents of *I. asprella* primarily include triterpenes, phenolic acids, polysaccharides, volatile oils, and others. *I. asprella* exhibits a wide range of pharmacological effects, including anti-inflammatory, antipyretic, analgesic, antimicrobial, antitumor, anti-complement, anti-ulcer, and anti-Alzheimer’s disease activities (Cai et al., 2024; Chen et al., 2024).

*I. asprella* is a commonly used medicinal material in the Lingnan region, including Guangdong, Guangxi, Fujian, etc. (Huang et al., 2023). Among these, *I. asprella* is produced throughout Guangdong Province, with a higher yield in the central region, and especially the counties in the suburbs of Guangzhou (Mei et al., 2020). *I. asprella* has been included in the list of medicinal herbs used by ethnic minorities such as Zhuang and Yao in Guangxi. There may be certain differences in quality, yield, and other aspects among *I. asprella* from different producing areas. In addition, during the production and processing of *I. asprella* medicinal materials, there are often chaotic phenomena such as confusion of sources, mixing of superior and inferior quality, and adulteration of fake or inferior varieties. For example, some unscrupulous traders in the market, to obtain higher profits, adulterate *I. asprella* with species such as *I. pubescens*, *I. rotunda*, and *I. wilsonii* (Zhang, 2024). These chaotic phenomena can easily affect the quality of *I. asprella* and hinder the development of the industry. Therefore, it is particularly important to conduct germplasm identification on different *I. asprella* germplasms.

SSR molecular marker identification is a common molecular biology technique that involves designing specific primers to amplify genomic DNA through PCR and then detecting the length polymorphism of the amplified products through electrophoresis or other methods to determine genetic differences among individuals. It is primarily used to analyze genetic variations within organisms and boasts advantages such as high polymorphism, codominant inheritance, good stability, and ease of operation. It is widely used in genetic diversity analysis, cultivar identification, kinship analysis, and gene mapping (Zhang et al., 2023). Currently, many researchers have utilized SSR molecular marker identification technology to conduct germplasm resource identification and genetic diversity investigations on different plant species (Bassil et al., 2020; Akin et al., 2016; Eyduran et al., 2016). For example, Zhang et al. (2023) conducted genetic analysis on 60 samples of *Pogostemon cablin* and developed an SSR molecular marker that can simply, rapidly, and effectively identify *P. cablin* samples, analyzing the genetic diversity of *P. cablin* resources. Wang et al. (2024) utilized SSR molecular markers to analyze the genetic diversity of 63 potato resources, clarifying the kinship among potato germplasm resources. As of now, there have been no reports on genetic diversity studies of the germplasm resources of *I. asprella*.

SSRs are widely distributed in genomes and exhibit abundant polymorphism, which originates from variations in the number of repeat units. Based on the polymorphism of SSRs, plant genomes can be analyzed and screened, providing valuable information for species identification, gene mapping, phylogenetic relationship identification, and so on. Kong et al. (2022) reported on the genome of *I. asprella*, providing invaluable information for SSR identification and development at the genomic level, as well as for germplasm identification and genetic diversity studies of *I. asprella*. Hence, based on the reported genomic data of *I. asprella*, this study conducted SSR identification and development at the genomic level. Furthermore, 192 pairs of SSR molecular markers were utilized to screen and evaluate 25 collected germplasm resources of *I. asprella*. To the best of our knowledge, this was the first study reporting the development and characterization of genomic SSRs in *I. asprella* and the genetic diversity analysis of its germplasm resources. The current study assessed the genetic relatedness among individuals and the level of genetic diversity within the population, providing a scientific basis for the subsequent conservation and utilization of *I. asprella*.

## 2 Materials and methods

### 2.1 Experimental materials

**Figure 1:**
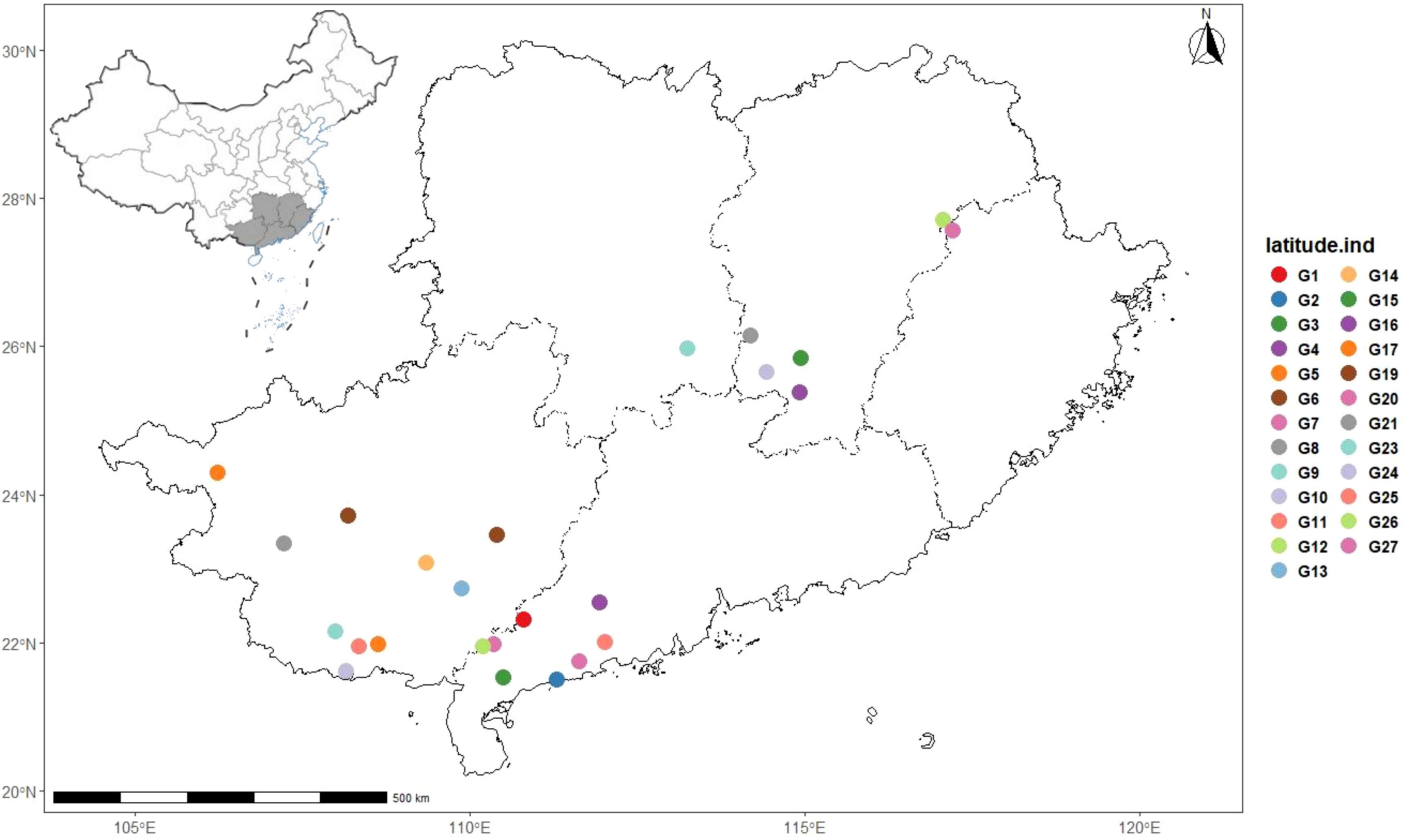
Distribution map of sampled germplasm resources of *I. asprella*.

**Figure 2:**
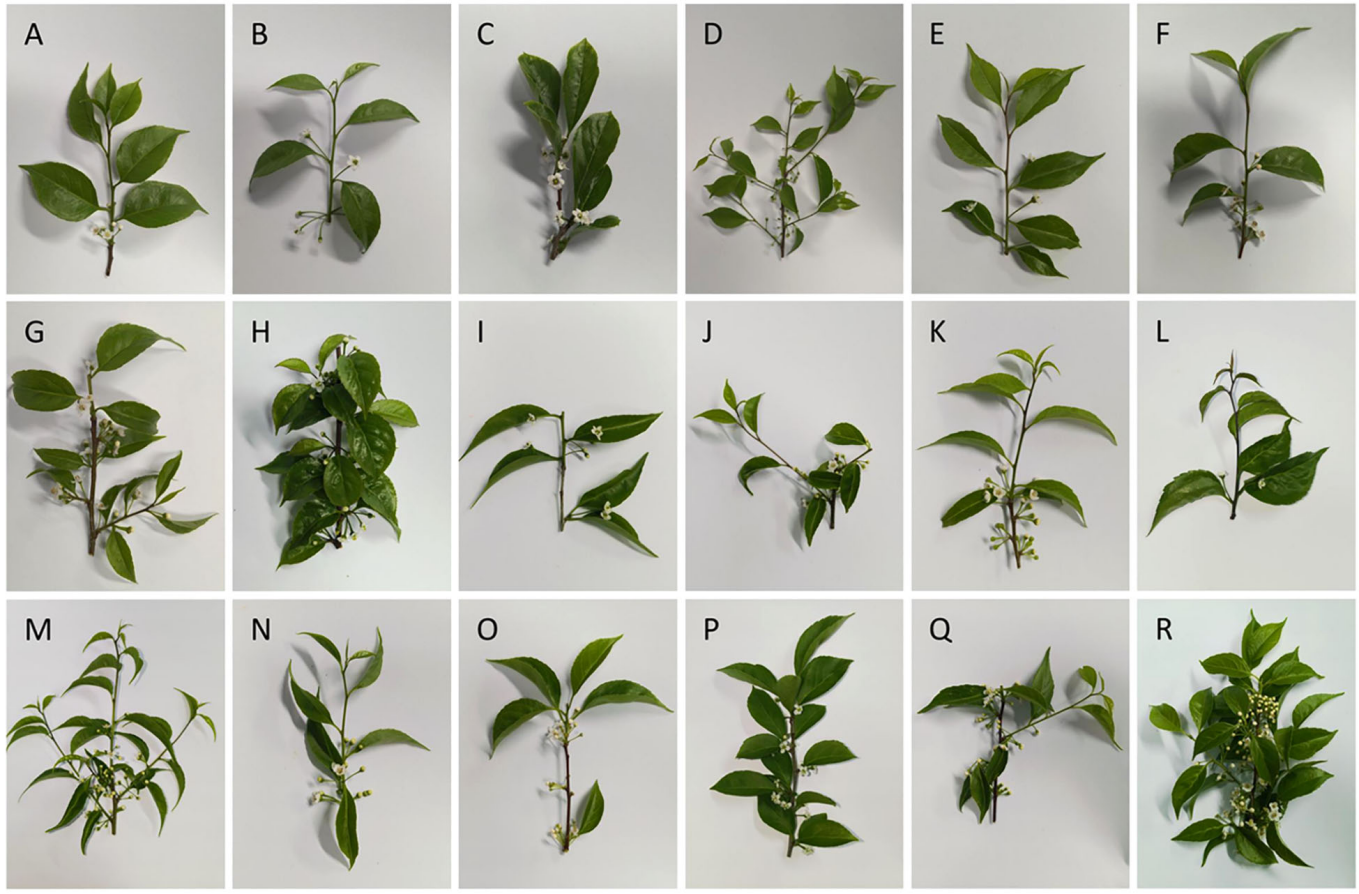
Morphological images of partial *I asprella* germplasm resources (flowers and leaves). **(A–R)** G1, G2, G3, G4, G5, G6, G8, G10, G11, G12, G14, G15, G17, G19, G20, G23, G24, G25.

The germplasm materials used in this study were collected from Guangdong, Guangxi, Hunan, Jiangxi, and Fujian, with a total of 25 germplasm resources (**Table 1**, **Figures 1**, **2**). Each germplasm resource contains 2~15 individual plants. All *I. asprella* germplasm resources were preserved in the planting management base (Yunfu City, Guangdong Province, China) and authenticated by Agronomist Yuquan Huang. For each germplasm, young leaves from at least three phenotypically consistent individual plants (disease- and pest-free) were selected for mixing, wrapped in aluminum foil, placed in ice packs, and stored in -80°C ultra-low temperature freezers for future use. For germplasms with fewer than three individual plants, leaves are collected from all available individuals and mixed as one sample.

**Table 1:** The origin of 25 *I. asprella* germplasm resources.

Name	Origin	Number of individual plants	Name	Origin	Number of individual plants
GM-G1	Xinyi City, Guangdong Province	10	GM-G2	Dianbai City, Guangdong Province	14
GM-G3	Gaozhou City, Guangdong Province	13	GM-G4	Xinfeng County, Jiangxi Province	10
GM-G5	Tianlin County, Guangxi Zhuang Autonomous Region	7	GM-G6	Pingnan County, Guangxi Zhuang Autonomous Region	8
GM-G7	Luchuan County, Guangxi Zhuang Autonomous Region	2	GM-G8	Tiandong County, Guangxi Zhuang Autonomous Region	9
GM-G9	Shangsi County, Guangxi Zhuang Autonomous Region	4	GM-G10	Jiangping Town, Fangchenggang City, Guangxi Zhuang Autonomous Region	10
GM-G11	Huashi Town, Fangchenggang City, Guangxi Zhuang Autonomous Region	5	GM-G12	Bobai County, Yulin City, Guangxi Zhuang Autonomous Region	7
GM-G13	Xingye County, Yulin City, Guangxi Zhuang Autonomous Region	2	GM-G14	Sanli Town, Guigang City, Guangxi Zhuang Autonomous Region	4
GM-G15	Shangyou County, Ganzhou City, Jiangxi Province	4	GM-G16	Yangchun City, Yangjiang City, Guangdong Province	3
GM-G17	Qinnan District, Qinzhou City, Guangxi Zhuang Autonomous Region	3	GM-G19	Mashan County, Nanning City, Guangxi Zhuang Autonomous Region	3
GM-G20	Yangxi County, Yangjiang City, Guangdong Province	2	GM-G21	Guidong County, Chenzhou City, Hunan Province	5
GM-G23	Zixing City, Chenzhou City, Hunan Province	10	GM-G24	Chongyi County, Ganzhou City, Jiangxi Province	15
GM-G25	Yangdong District, Yangjiang City, Guangdong Province	12	GM-G26	Zixi County, Fuzhou City, Jiangxi Province	3
GM-G27	Huaqiao Township, Guangze County, Fujian Province	3			

### 2.2 Extraction of genomic DNA

In the experiment, 25 samples of total DNA were extracted from the leaves of the germplasm resources of *I. asprella* using the genomic DNA extraction kit [Tiangen Biochemistry Technology (Beijing) Co.]. The quality of the DNA was detected by agarose gel electrophoresis, and the quality and concentration of the DNA were also determined by using NanoDrop ONE Ultra-micro UV Spectrophotometer. The OD260/OD280 value of 1.8~2.0 was used as a reference, and the samples that did not meet the standard were re-extracted, and the final sample DNA was diluted to 50 ng/μL and stored in a refrigerator at -20°C for spare use.

### 2.3 Genome-wide identification and development of SSR

According to the reference genome of *I. asprella* reported by Kong et al. (2022), SSR loci in the genomic sequence were analyzed using MISA 2.0 software. The MISA analysis parameters were as follows: (1) Definition of microsatellites (unit size/minimum number of repeats): (1/20), (2/6), (3/5), (4/5), (5/5), (6/5); (2) Maximal number of bases interrupting two SSRs in a compound microsatellite: 100. The Primer 3 program were used to design primers for genotyping SSR loci. The primer design parameters were as follows: (1) Primer sequence length of 18 to 22 bp; (2) Amplified product length of 110 to 350 bp; (3) Annealing temperature (Tm value) of 50°C to 60°C; and (4) Amplified product GC content of 40% to 60%. The criteria for screening SSR loci were: (1) Exclude loci for which primers cannot be designed based on the above parameters; (2) Exclude loci with single-nucleotide repeat units and composite repeat units; (3) Exclude loci where the repeat units consist entirely of G/C bases; (4) Exclude SSR markers with identical upstream or downstream primer sequences; (5) The priority for screening repeat units is 3n, 4n, 5n, 6n, and 2n in sequence; (6) Select loci based on the number of repeats from highest to lowest; (7) Preferentially select loci from different gene sequences; (8) Include loci with different repeat units as much as possible.

### 2.4 Synthesis and screening of typing primers

From the SSR markers that meet the above criteria, 192 pairs were randomly selected for primer screening experiments. Primer synthesis utilized an adapter method, where a 21 bp adapter sequence was added to the upstream primer during synthesis. Firstly, based on our preliminary phenotype observation at the planting management base, we selected 5 samples (G1: thick leaves; G3: hairy stems and leaves, thick leaves; G10: large leaves; G13, G14: narrow and elongated leaves) with significant phenotypic and genetic background differences and obtained 15 pairs of SSR primers with good polymorphism (Polymorphism Information Content, PIC > 0.25) in this study. Then, we used these 15 pairs of primers to detect and genotype 25 samples.

### 2.5 PCR amplification and fluorescent PCR amplification systems

The 15 pairs of primers for SSR capillary electrophoresis are listed in **Table 2**. In this study, the M13 universal junction sequence (TGTAAAACGACGGGCCAGT) was added to the 5′ direction of the F primer of each primer pair, and three different kinds of fluorescent markers were chosen to be used, namely, FAM, HEX, and TAMRA, to complete the synthesis of M13 junction sequences carrying different fluorescent moieties. Then the PCR products carrying fluorescence were detected by fluorescence electrophoresis using a DNA sequencer, ABI3730xl, and the initial data of the experiment were band-typed using GeneMarkerv 2.2.0 software.

**Table 2:** Marker information of 15 SSR loci in *I. asprella*.

Loci	Repeat unit	Forward primer	Reverse primer	Product size	PIC value
LAC091	(GA)8	GACTGGTGTAGGGCTGGAAC	CAGTCACCGGCTGTCTCTTT	254	0.586
LAC187	(AG)11	TGGGACTTGCACAGTGAGAG	AGGCAGAACCAAAGTACGAA	185	0.586
LAC076	(TC)9	AGCTGTCCTCCAGACCATCA	CATCCTCCCAATCCGACACC	259	0.596
LAC097	(CT)14	ATTGCAGCCACCTCTTTCGA	AGAGGTGTGCGTGCTTACTC	138	0.596
LAC089	(TC)8	TCTCATTCTCCCGGTGCAAC	TGCCACGAGTTCTCTAAGCC	274	0.61
LAC052	(TC)11	ACGCTGCAAGATACACGACA	CGGAGATGAGTGGTGCTGTT	239	0.645
LAC113	(GAC)7	TCGGCGTGTTAATTGGACCA	TTCCGAATCCGACCCAACAG	191	0.666
LAC082	(GAA)10	GACCACTGACGAGAACAGCA	GCGAGCCTCTTTCCTCAACT	280	0.72
LAC147	(AG)17	ACAACTGGGTGTGGTGTTGT	TGATGAGCCTTCTTGGAGCG	197	0.72
LAC020	(CA)13	TTTCTGTGGTGACGCCGATC	AGAAGCTTACACCAAGGCGT	278	0.745
LAC137	(TC)12	AGCCAAACCCACGCTAAAGA	TTGTTTGGCTGCCTTGGTTG	168	0.745
LAC155	(AG)18	CGGGAGTCTAGTCTAGGGCA	GAGGTGCTTGAAGTTTCGCG	280	0.768
LAC073	(AG)8	TCCTTCCCATTTCGACCCTT	AGTTTAGGGCGCGACCTATG	163	0.772
LAC125	(GT)7	GTGGAGGAGGTGGTTCTGTG	GAGACCCACTTCAGCAACGA	154	0.772
LAC140	(AG)18	AGCAAGGAAGGTGGAGAGAA	TGTCAACATCCGGTCCCTTT	168	0.798

0<PIC<0.25 indicates low polymorphism, 0.25<PIC<0.5 indicates moderate polymorphism, and PIC>0.5 indicates high polymorphism.

PCR reaction conditions: pre-denaturation at 95°C for 5 min; denaturation at 95°C for 30 s, annealing at a gradient of 62~52°C for 30 s, extension at 72°C for 30 s, running for 10 cycles, with a decrease of 1°C in each cycle; denaturation at 95°C for 30 s, annealing at 52°C for 30 s, extension at 72°C for 30 s, running for 25 cycles; extension at 72°C for 20 min; and finally, the PCR reaction was carried out in a PCR machine. 4°C for storage. Fluorescence PCR reaction conditions: pre-denaturation at 95°C for 5 min; denaturation at 95°C for 30 s, annealing at a gradient of 62~52°C for 30 s, extension at 72°C for 30 s, running for 10 cycles; denaturation at 95°C for 30 s, annealing at 52°C for 30 s, extension at 72°C for 30 s, run for 25 cycles; extension at 72°C for 20 min; the final PCR product was placed in a refrigerator at 4°C for storage. SSR-PCR amplification was performed using a 10 μL reaction system: 5 μL 2×Taq PCR Master Mix (Genetech), 1 μL Mix primer, 1 μL DNA Template (50~200 ng), and 3 μL ddH_2_O to make up the 10 μL PCR reaction system.

### 2.6 Amplification product identification

Fluorescent PCR products were identified by agarose gel electrophoresis, and PCR bands were detected using the electrophoresis results. Single bands of matching size were selected and quantified against the concentration of the DNA Marker, and all products were diluted to the same concentration range and tested on the machine.

### 2.7 Data reading and processing

The raw data in.fsa format were exported from the ABI 3730xl instrument, categorized and filed according to the detected loci, and then imported into the GeneMarker analysis software for genotypic data reading, and exported the Excel genotypic raw data and PDF genotyping peak map files according to the loci names, respectively. When analyzing the loci, the calculation of parameters such as the number of alleles (Na), the number of effective alleles (Ne), the Shannon information index (I), the observed heterozygosity (Ho), the expected heterozygosity (He), the average expected heterozygosity (uHe), and the fixation index (F) was completed on the GenAIex6.5 software and calculated to obtain the F-Statistics. The degree of genetic differentiation and calculation of genetic distance were performed, and PCoA and AMOVA analyses were conducted using GenAlEx software (version 6.501). For cluster analysis, the Phylip software was used to construct an evolutionary tree by UPGMA for the population of *I. asprella*, and Structure 2.3.4 software was used to analyze the genetic structure of the population.

## 3 Results

### 3.1 Number and types of SSR loci in the *I. asprella* genome

**Figure 3:**
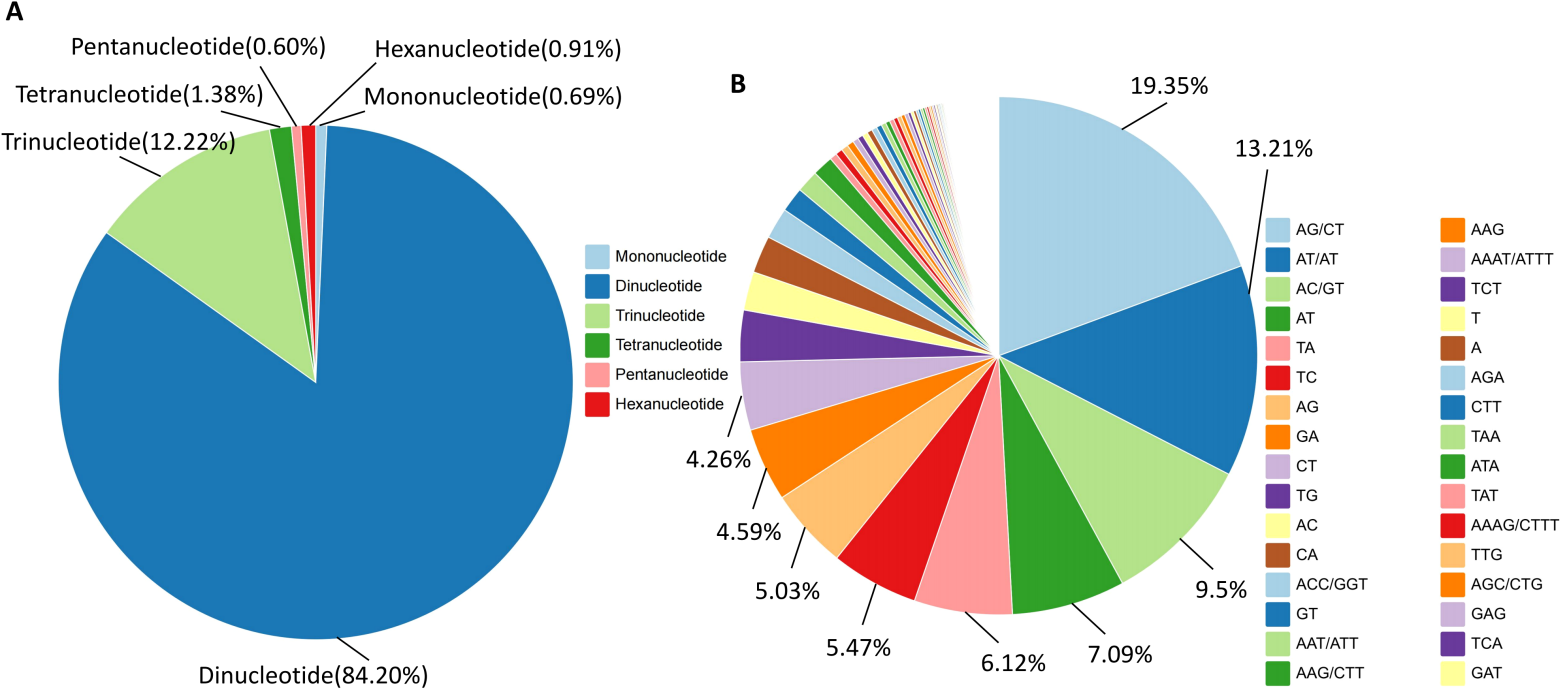
Discovery of SSR loci in the whole genome of *I. asprella*. **(A)** The distribution of SSR loci with different types of repeats in the genome of *I. asprella*; **(B)** Types and proportions of SSR repeat elements in the genome of *I. asprella*.

A total of 137,443 SSR loci were detected across the whole genome of *I. asprella*. Six types of SSRs were obtained, including mononucleotide, dinucleotide, trinucleotide, tetranucleotide, pentanucleotide, and hexanucleotide repeats. The dinucleotide and trinucleotide repeats were dominant, with dinucleotide repeat motifs accounting for 84.20% of the total markers and trinucleotide repeat motifs accounting for 12.22%. Pentanucleotide repeats had the smallest proportion, at 0.60% (**Figure 3A**). AG/CT was the most abundant repeat motif, with a total of 53,202 occurrences. The next most common motifs were AT/AT and AC/GT, with 36,311 and 26,104 occurrences respectively. All other repeat motif types had fewer than 20,000 occurrences (**Figure 3B**; **Supplementary Table S1**).

### 3.2 Screening of SSR primers for *I. asprella*

Based on the reported genome sequencing results of *I. asprella* (Kong et al., 2022), the MISA online software was utilized to analyze the genome information of *I. asprella*, resulting in the identification of SSR loci. SSR primers were designed using Primer Premier 3.0 software, and 192 pairs of primers were selected for synthesis and screened for polymorphism. In this study, five germplasms of *I. asprella* (G1, G3, G10, G13, G14) were selected based on factors such as significant genetic background differences and morphological variations. PCR amplification was performed on the DNA of these five germplasms using the 192 pairs of primers. The PCR amplification products were detected using capillary electrophoresis, and the primers were screened twice, ultimately selecting 45 polymorphic primers with large peak differences. The primer quality was evaluated based on the polymorphism information content (PIC) values and agarose gel electrophoresis banding patterns, and 15 pairs of primers were recommended for population genotyping (**Table 2**, **Supplementary Figure S1**).

**Figure 4:**
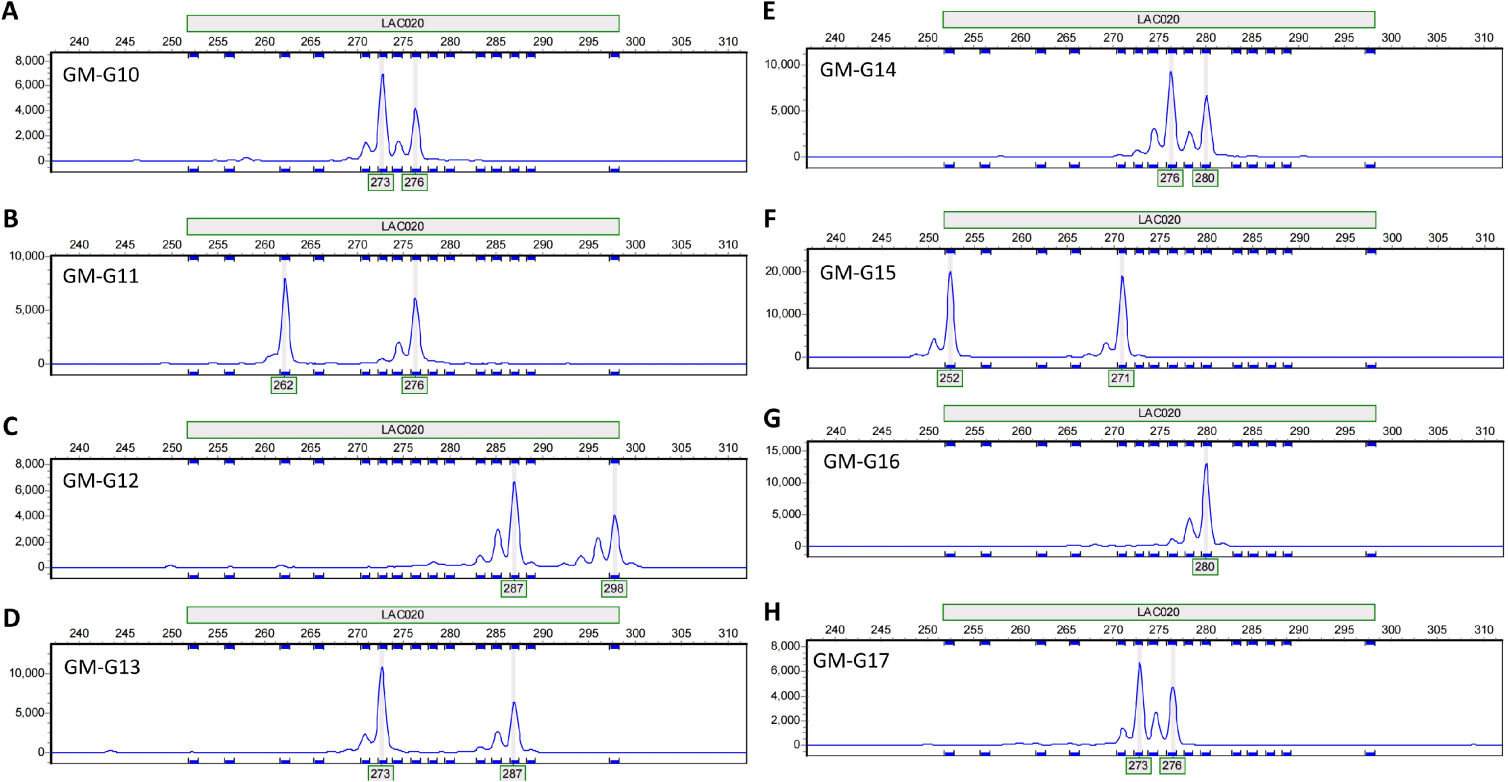
Allelic variations detected by primer LAC020 in eight *I. asprella* germplasms. **(A–H)** The allelic variations detected by primer LAC020 in GM-G10, GM-G11, GM-G12, GM-G13, GM-G14, GM-G15, GM-G16, and GM-G17, respectively.

Using the selected 15 pairs of polymorphic primers, PCR amplification was performed on the DNA of various *I. asprella* germplasms. The PCR products were then subjected to capillary electrophoresis detection. Based on the capillary electrophoresis results, rapid identification of different *I. asprella* germplasms at the genetic level could be achieved. For instance, primer LAC020 amplified product fragments of different sizes in eight distinct *I. asprella* germplasms, GM-G10, GM-G11, GM-G12, GM-G13, GM-G14, GM-G15, GM-G16, and GM-G17 (**Figure 4**), indicating that primer LAC020 can be used for the identification of *I. asprella* germplasm resources, and SSR primers exhibit good polymorphism.

### 3.3 Genetic diversity parameters of SSR loci in the *I. asprella* genome

After calculation and analysis using GenAlEx version 6.501 software, the genetic diversity indices of all the *I. asprella* germplasms at 15 SSR loci were obtained. The amplification results of 25 *I. asprella* germplasms at these 15 loci are shown in **Table 3**. The number of alleles (Na) ranged from 5 (LAC113) to 15 (LAC052), with an average of 9.47 alleles and an average of 5.231 effective alleles (Ne). The closer the value of effective alleles is to the absolute value of the number of alleles, the more evenly distributed the alleles are in the population (Luo, 2009). Our data indicated that the number of alleles was higher than the number of effective alleles, suggesting that the distribution of alleles at SSR loci in the *I. asprella* genome was uneven.

**Table 3:** Genetic diversity parameters of 15 SSR loci in *I. asprella*.

locus	Na	Ne	I	Ho	He	F	PIC	Prob	Signif
LAC020	13	8.224	2.3	0.72	0.878	0.18	0.867	0.193	ns
LAC052	15	8.993	2.407	0.8	0.889	0.1	0.879	0.227	ns
LAC073	11	9.328	2.314	0.68	0.893	0.238	0.883	0.241	ns
LAC076	13	6.345	2.177	0.52	0.842	0.383	0.829	0	***
LAC082	8	4.721	1.784	0.458	0.788	0.418	0.764	0	***
LAC089	6	2.7	1.287	0.48	0.63	0.238	0.592	0.001	***
LAC091	7	3.571	1.503	0.52	0.72	0.278	0.676	0.175	ns
LAC097	8	3.173	1.486	0.48	0.685	0.299	0.65	0.001	***
LAC113	5	4.281	1.527	0.56	0.766	0.269	0.73	0.006	**
LAC125	8	4.255	1.689	0.682	0.765	0.109	0.735	0.351	ns
LAC137	11	4.579	1.831	0.72	0.782	0.079	0.753	0	***
LAC140	12	5.73	2.055	0.625	0.825	0.243	0.806	0	***
LAC147	11	4.545	1.829	0.68	0.78	0.128	0.751	0.262	ns
LAC155	9	4.325	1.789	0.48	0.769	0.376	0.747	0	***
LAC187	5	3.698	1.422	0.28	0.73	0.616	0.684	0	***
Mean	9.46667	5.2312	1.82667	0.579	0.7828	0.2636	0.7564		
St Dev	3.09069	2.08678	0.35466	0.13693	0.07476	0.14349			

Na, observed allele; Ne, effective allele; I, Shannon’s index; Ho, observed heterozygosity; He, expected heterozygosity; F, fixation index, an index that assesses the degree of deviation of the actual observations from the theoretical values; PIC, polymorphic information index; Prob, P-value; Signif, significance (ns denotes non-significant, i.e., the population conforms to the HWE; * denotes significant difference P< 0.05, ** indicates significant difference P<0.01, *** indicates significant difference P<0.001).

Heterozygosity is one of the important indicators for assessing population genetic diversity, as it is calculated based on the gene frequency of each allele and is not easily affected by sample size, thereby more accurately reflecting the level of genetic diversity in the population (Qin et al., 2014). Among the 15 loci, the observed heterozygosity (Ho) ranged from 0.28 (LAC187) to 0.8 (LAC052), and the expected heterozygosity (He) ranged from 0.63 (LAC089) to 0.893 (LAC073), indicating differences in heterozygosity among different loci. Among the 15 loci, the average Ho of all loci was lower than the average He, with average values of 0.579 and 0.783, respectively. This suggested that there may be genetic variation in the experimental pedigrees, with good genetic diversity and partial changes in genotype frequencies within the population.

According to the definition of the fixation index (F), when there is an excess of homozygotes in the population, F>0; conversely, when there is an excess of heterozygotes, F<0 (Botstein et al., 1980). In this study, the fixation index (F) for all 15 loci was greater than 0, indicating an excess of homozygotes in the population.

The polymorphism information content (PIC) for the 15 loci in this study ranged from 0.592 to 0.883, with an average of 0.7564, all of which were highly polymorphic loci (**Table 2**). This indicated that the SSR markers selected in this study had a relatively rich distribution of polymorphism and could effectively analyze subsequent genetic diversity.

Fis, also known as the Hardy-Weinberg disequilibrium index (D), indicates the degree of deviation from random mating in a population and can be used to test for the deficiency or excess of heterozygotes in a population. When D>0, it indicates an excess of heterozygotes; when D<0, it indicates a deficiency of heterozygotes; and when D approaches 0, it indicates that the gene distribution tends to equilibrium (Fan et al., 2023). In this study, 11 loci (LAC020, LAC073, LAC076, LAC082, LAC089, LAC091, LAC097, LAC113, LAC140, LAC155, LAC187) exhibited an excess of heterozygotes, while 4 loci (LAC052, LAC125, LAC137, LAC147) showed a deficiency of heterozygotes (**Table 4**).

**Table 4:** Inbreeding coefficients and gene flow for 15 primer pairs in *I. asprella*.

Locus	Fis	Fit	Fst	Nm
LAC020	0.029	0.245	0.223	0.874
LAC052	-0.045	0.065	0.105	2.125
LAC073	0.172	0.279	0.129	1.69
LAC076	0.245	0.448	0.269	0.679
LAC082	0.341	0.441	0.153	1.388
LAC089	0.089	0.284	0.215	0.913
LAC091	0.126	0.233	0.122	1.8
LAC097	0.102	0.287	0.206	0.963
LAC113	0.125	0.261	0.155	1.36
LAC125	-0.147	0.111	0.225	0.861
LAC137	-0.124	0.036	0.142	1.51
LAC140	0.036	0.193	0.163	1.286
LAC147	-0.142	0.186	0.287	0.62
LAC155	0.18	0.36	0.22	0.889
LAC187	0.506	0.641	0.274	0.664
Mean	0.099	0.271	0.192	1.175
SE	0.047	0.041	0.015	0.119

Fis, the inbreeding coefficient within the group; Fit, overall inbreeding coefficient; Fst, genetic differentiation coefficient; Nm, gene flow (Nm=0.25 (1- Fst)/Ft).

The range of Fst values is from 0 to 1. A maximum value of 1 indicates complete differentiation between two populations, while a minimum value of 0 indicates no differentiation between populations. In practical research, Fst values between 0 and 0.05 indicate very little genetic differentiation between populations and can be ignored; values between 0.05 and 0.15 indicate moderate genetic differentiation; values between 0.15 and 0.25 indicate substantial genetic differentiation; and values above 0.25 indicate significant genetic differentiation between populations (Zhu et al., 2017). In this study, the mean Fst value for the population differentiation rate of *I. asprella* was 0.192 (**Table 4**), indicating substantial genetic differentiation at different SSR loci in the *I. asprella* population genome.

Gene flow (Nm) is negatively correlated with the genetic differentiation coefficient (Fst) and refers to the impact of genes carried by individuals on population genetic variation during migration. When Nm>1, it indicates that there is gene flow between different populations, leading to increased genetic similarity and thus slowing down the genetic differentiation between populations. When Nm<1, it means that the effect of gene flow is relatively weak, and genes between populations are difficult to effectively spread and diffuse. Gene flow is not sufficient to offset genetic drift within populations, in which case genetic drift plays a major role in genetic differentiation (Zhao et al., 2023; Slaktin, 1987). In this study, the range of Nm at different loci in the *I. asprella* population genome was 0.620 to 2.125, with an average Nm of 1.175 (**Table 4**). There were 8 loci with Nm<1 (LAC020, LAC076, LAC089, LAC097, LAC125, LAC147, LAC155, LAC187) and 7 loci with Nm>1 (LAC052, LAC073, LAC082, LAC091, LAC113, LAC137, LAC140), indicating that there might be some gene flow in the *I. asprella* population.

### 3.4 Genetic diversity of *I. asprella* germplasm resources

**Figure 5:**
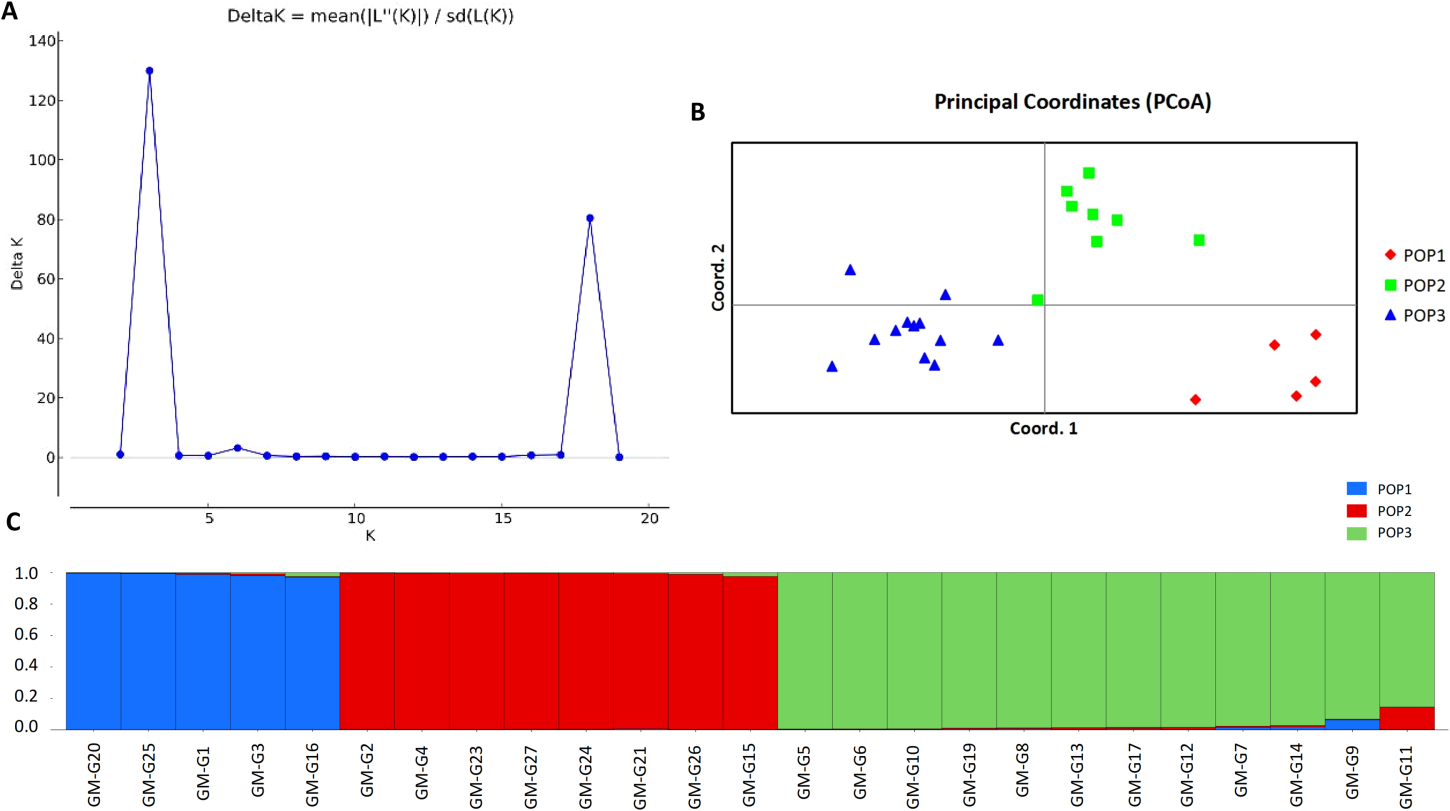
Genetic structure analysis of 25 germplasm samples of *I. asprella.* **(A)** The K value variation chart drawn by the ΔK method of structure analysis; **(B)** Principal coordinate analysis of 25 samples; **(C)** The structure results of 25 samples at K=3. Different colors represent different gene banks, with the horizontal axis representing the germplasm number of *I. asprella* and the vertical axis representing the proportion of a certain germplasm to a certain population component.

Using the 15 molecular markers, the population structure of 25 samples was evaluated (**Figure 5A**). Based on the principle of maximum likelihood, the optimal K value was determined to be 3, allowing the 25 samples to be divided into three subpopulations. Principal Coordinates Analysis (PCoA) was utilized to analyze the genetic differentiation among the three populations mentioned above (**Figure 5B**). The results indicated that pop2 and pop3 were genetically closer, while pop1 was genetically distant from the other populations. The samples within each population were relatively concentrated and not dispersed, and the differences among the three populations were apparent. As shown in **Figure 5C**, all the tested germplasms had three possible gene pools and gene flow in the genus of *I. asprella* occurred only in a few individuals, with low levels of gene flow observed in most of the germplasms. According to the results of the Analysis of Molecular Variance (AMOVA) (**Table 5**), 18% of the genetic variation in *I. asprella* existed among populations, while 82% existed within individuals. This indicated that genetic variation was present not only within populations but also within individuals, with individual variation being the primary source of total variation.

**Table 5:** Analysis of molecular variance (AMOVA) of populations.

Source	df	SS	MS	Est. Var.	%
Among Pops	2	49.268	24.634	1.168	18%
Among Indiv	22	138.913	6.314	1.017	16%
Within Indiv	25	107	4.28	4.28	66%
Total	49	295.18		6.465	100%

Source: source of variation; df: degrees of freedom; SS: total variance; MS: mean square deviation; Est. Var.: estimated value of variance; %: percentage of variation; Among Pops: between populations; Among Indiv: between individuals; Within Indiv: intra-individual, intra-individual variance refers to genetic differences caused by heterozygous alleles, the size of which correlates with the number of individual. The size of intra-individual variation is related to the number of heterozygous loci, i.e., the genetic diversity of individuals.

Using GenAlex6.5 software to analyze capillary electrophoresis results, based on the fragment sizes amplified at different loci among different germplasms of *I. asprella*, 25 germplasms of *I. asprella* can be divided into three populations (**Table 6**). Within each population, the number of observed alleles (Na) was higher than the number of effective alleles (Ne), indicating an uneven distribution of alleles within the populations. Shannon’s information index (I) can be used to estimate genetic differentiation within populations. The larger the index, the greater the genetic diversity and the higher the degree of population differentiation (Zhang, 2008). In this study, the Shannon’s information index (I) for pop1 was 0.9796; for pop2, it was 1.4750; and for pop3, it was 1.2982. This indicated that pop2 had the highest genetic diversity, followed by pop3, and pop1 had the lowest genetic diversity. It was inferred that there were differences in the level of genetic diversity among the populations. The fixation index (F) for all three populations is positive, and the Ho is lower than the He. Therefore, the number of heterozygotes within the genotypes of the three populations was less than theoretically expected, indicating the presence of homozygous excess.

**Table 6:** Genetic diversity among populations of *I. asprella* germplasm resources.

Pop		Na	Ne	I	Ho	He	F
POP1	Mean	3.266666667	2.465349723	0.979626366	0.56	0.562416667	0.058265853
	SE	0.206251503	0.178732861	0.068621411	0.080946779	0.032948478	0.11967175
POP2	Mean	5.666666667	4.080697916	1.475065046	0.614285714	0.718465372	0.15720606
	SE	0.566246343	0.474598565	0.095482599	0.056167267	0.024078099	0.069309665
POP3	Mean	5.6	3.155847111	1.298186069	0.565151515	0.640912917	0.117367465
	SE	0.505210941	0.303252173	0.099081753	0.047860793	0.033680029	0.066085965
Total	Mean	4.844444444	3.233964917	1.25095916	0.57981241	0.640598319	0.110946459
	SE	0.306422095	0.216758909	0.058844786	0.035862221	0.019727609	0.050290941

Na, observed allele; Ne, effective allele; I, Shannon’s index; Ho, observed heterozygosity; He, expected heterozygosity; F, fixation index.

### 3.5 Analysis of population genetic structure of *I. asprella* germplasm resources

**Figure 6:**
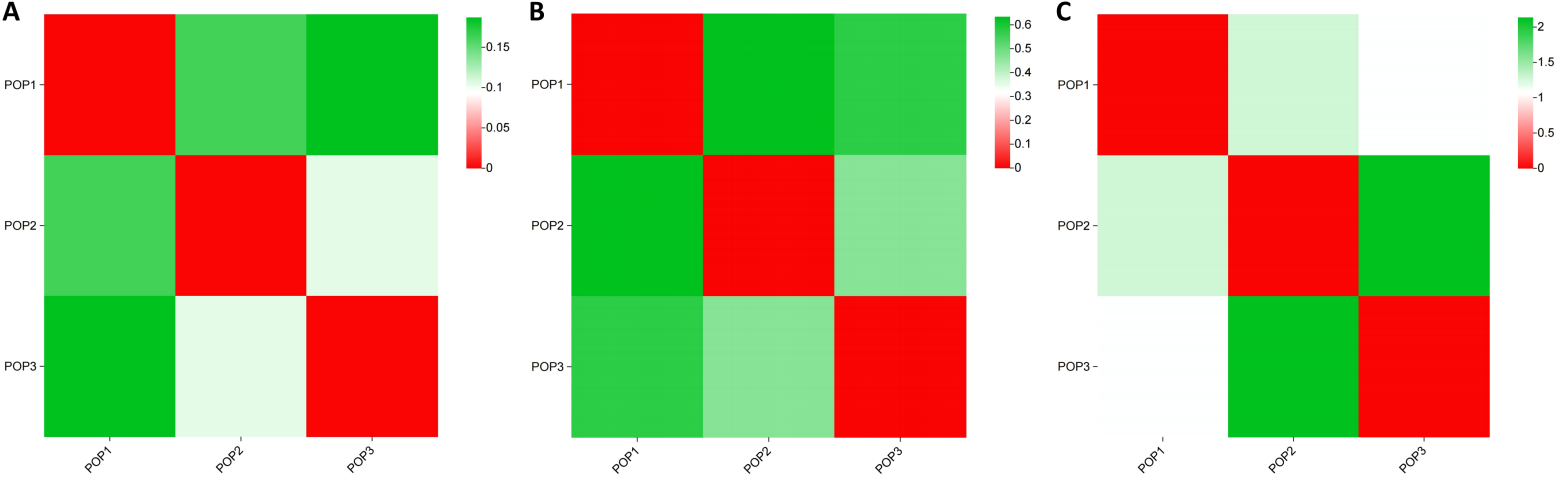
Analysis of population genetic structure. **(A)** Genetic differentiation coefficient; **(B)** Genetic distance; **(C)** Gene flow.

**Figure 7:**
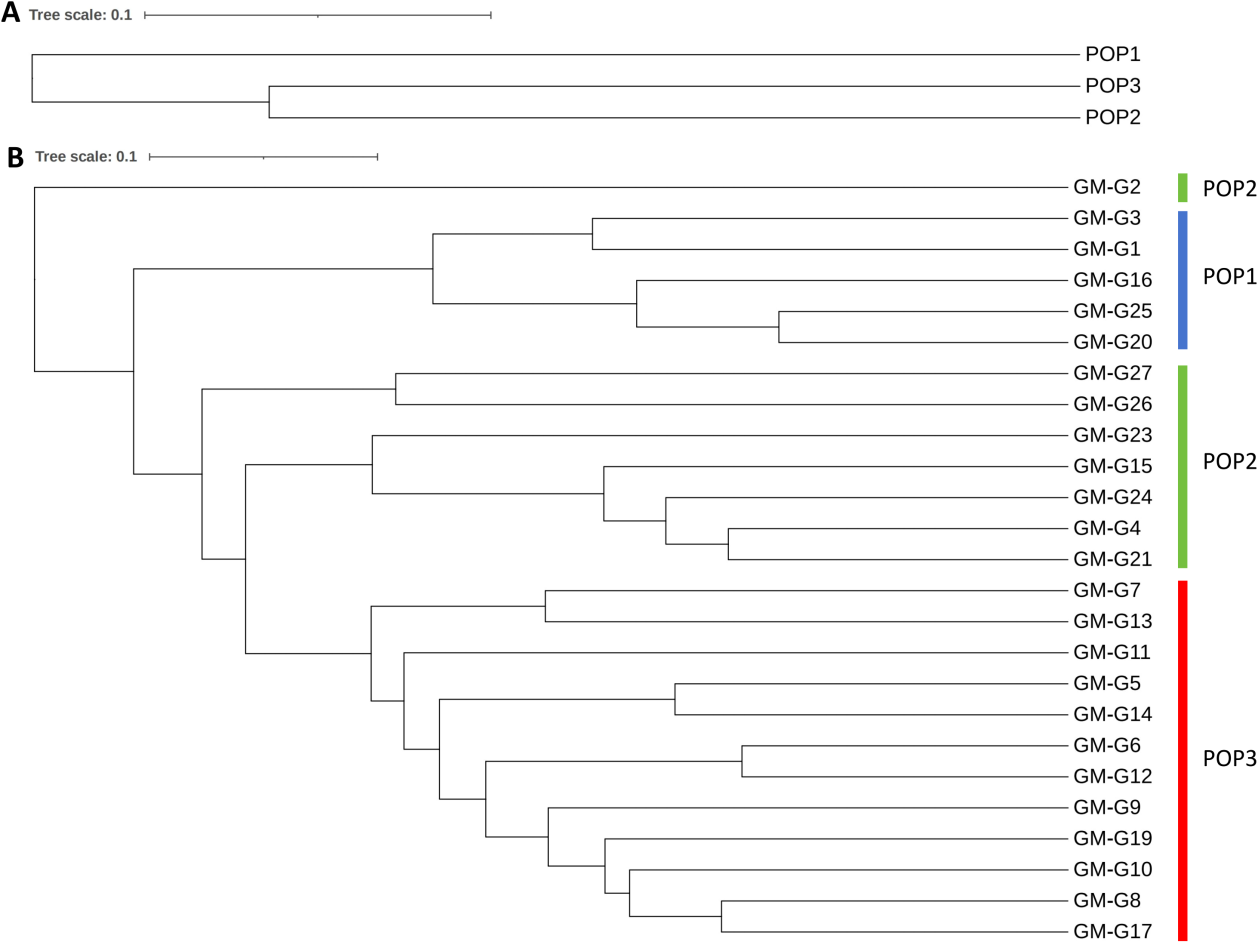
UPGMA clustering results. **(A)** UPGMA clustering results of three populations; **(B)** UPGMA clustering results of 25 *I. asprella* germplasms.

**Figure 8:**
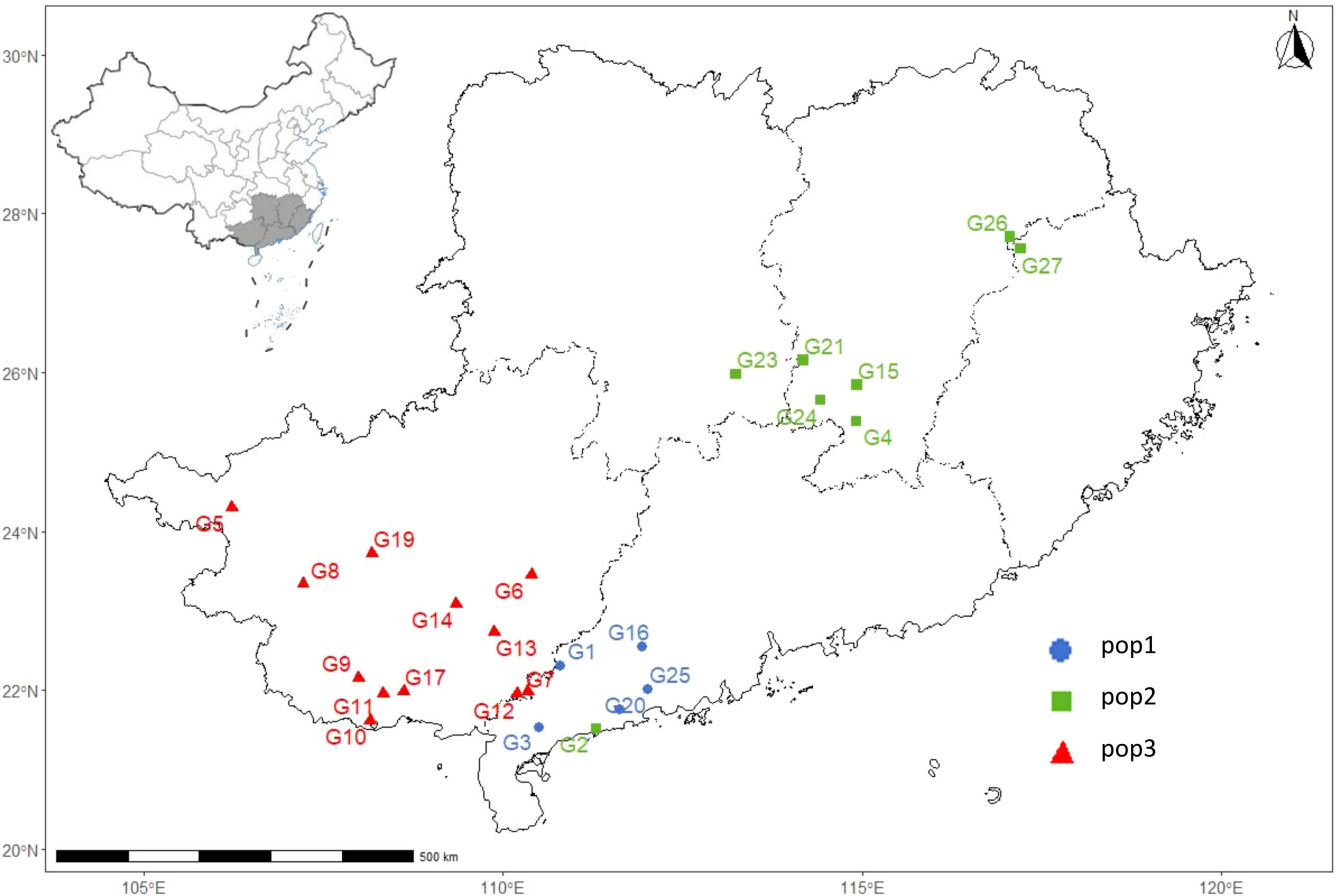
Geographical distribution map of 25 *I. asprella* germplasms that can be divided into 3 subgroups.

We calculated the genetic differentiation coefficient, genetic distance, and gene flow among populations to explore their genetic relationships (**Figure 5**; **Supplementary Tables S2**, **S3**). The analysis of the genetic differentiation coefficient revealed that the Fst values among populations ranged from 0.105 to 0.187 (**Figure 6A**). The smallest genetic differentiation was observed between pop2 and pop3 (Fst=0.105), followed closely by pop1 and pop2 (Fst=0.163). The largest genetic differentiation coefficient was found between pop1 and pop3, with an Fst value of 0.187. The genetic distances among the three populations were calculated using PowerMarker, with the maximum distance being 0.634173 (between pop1 and pop2) and the minimum being 0.468505 (between pop2 and pop3) (**Figure 6B**; **Supplementary Table S2**). The analysis of gene flow (Nm) among the three populations showed that Nm values were all greater than 1, with the maximum being 2.137 (between pop2 and pop3), followed by 1.283 (between pop1 and pop2), and the smallest being 1.088 (between pop1 and pop3) (**Figure 6C**; **Supplementary Table S3**). Both the genetic differentiation coefficient, genetic distance, and gene flow indicated that the genetic relationship between pop2 and pop3 was the closest. Using the Phylip software, an evolutionary tree was constructed based on the UPGMA method for all the germplasm of *I. asprella* in this study (**Figure 7**). The 25 germplasms could be divided into three subgroups. Pop2 and Pop3 clustered into one branch, indicating that these two populations were closely related and relatively distant from the other population, Pop1. The germplasm resources of *I. asprella* in Pop1 included GM-G1, GM-G3, GM-G16, GM-G20, and GM-G25; those in Pop2 included GM-G2, GM-G4, GM-G15, GM-G21, GM-G23, GM-G24, GM-G26, and GM-G27; and those in Pop3 included GM-G5, GM-G6, GM-G7, GM-G8, GM-G9, GM-G10, GM-G11, GM-G12, GM-G13, GM-G14, GM-G17, and GM-G19 (**Figure 8**).

## 4 Discussion

In this study, a total of 137,443 SSR loci were obtained from the whole genome sequence analysis of *I. asprella*, indicating a wide distribution of SSRs in its genome. Previous studies have shown that trinucleotide to hexanucleotide SSR markers can better detect differences in allele length at various loci compared to mononucleotide and dinucleotide SSR markers (Qin et al., 2022). In this study, a total of 15 polymorphic primers were ultimately selected, including 13 dinucleotide primers and 2 trinucleotide primers (**Table 2**), which could effectively detect alleles. This was inconsistent with the previous conclusion, possibly due to differences in species. Additionally, the 15 pairs of polymorphic primers screened in this study were all highly polymorphic loci (**Table 2**), ensuring subsequent genetic diversity analysis of the *I. asprella* population and reflecting the diversity of germplasm resources of *I. asprella*.

Parameters such as alleles (Na), effective alleles (Ne), observed heterozygosity (Ho), expected heterozygosity (He), and polymorphism information content (PIC) can reflect the level of genetic diversity within a population, and their numerical values are correlated with gene diversity (Tang et al., 2024). In this study, SSR molecular marker technology was used to classify 25 germplasm resources of *I. asprella* into three populations, with significant genetic differentiation among the populations. Additionally, the average number of Na across the three *I. asprella* populations was 4.844, the average number of Ne was 3.234, the average Ho was 0.580, and the average He was 0.641 (**Table 6**). These parameters collectively indicated the presence of significant genetic differentiation and a high level of genetic diversity within the *I. asprella* populations.

Among the three populations of *I. asprella*, the fixation index (F) was positive, and the observed heterozygosity (Ho) was lower than the expected heterozygosity (He), indicating a deficiency of heterozygotes and an excess of homozygous individuals. The STRUCTURE and PCoA analysis resulted in the division of the populations into three groups (**Figure 5**), which was generally consistent with the results of the UPGMA cluster analysis (**Figure 7**) among the individuals of *I. asprella*. The three populations largely corresponded to their geographical distribution (**Figure 8**), indicating genetic differentiation among the populations. This differentiation may be attributed to geographical isolation or limited gene flow. However, gene flow among POP1, POP2, and POP3 was greater than 1 (**Figure 6C**), suggesting the presence of genetic exchange between these three populations. Gene flow is not sufficient to offset genetic drift within populations, in which case genetic drift plays a major role in genetic differentiation (Zhao et al., 2023; Slaktin, 1987).

After analyzing all the germplasm resources of *I. asprella* using Shannon’s Information Index (I), we found that among the three populations of *I. asprella*, pop2 exhibited the highest level of genetic diversity. It encompassed eight germplasm resources: GM-G2, GM-G4, GM-G15, GM-G21, GM-G23, GM-G24, GM-G26, and GM-G27, originating from Dianbai City, Guangdong Province; Xinfeng County, Jiangxi Province; Shangyou County, Jiangxi Province; Guidong County, Hunan Province; Zixing City, Hunan Province; Chongyi County, Jiangxi Province; Zixi County, Jiangxi Province; and Guangze County, Fujian Province, respectively. Pop3 ranked second in terms of genetic diversity, with all its germplasm resources sourced from Guangxi Zhuang Autonomous Region. In contrast, Pop1 displayed the lowest level of genetic diversity, with its germplasm derived solely from Guangdong Province (**Table 1**). This suggested a certain correlation between geographic distance and genetic diversity in *I. asprella*, with populations from different provinces exhibiting higher levels of genetic diversity and populations from the same province showing relatively lower levels. Additionally, based on the UPGMA clustering results (**Figure 7**), we found that in the pop2 group, the germplasm from Jiangxi Province and Hunan Province were closer to each other, while the GM-G2 from Dianbai City, Guangdong Province, had relatively large genetic differences with others. This further confirmed the inference that the genetic diversity of the *I. asprella* germplasm and the relationship between germplasm resources may be affected by geographic distance. On the other hand, the germplasm in pop2 had similar affinities (except for G2), even though they came from different geographical areas, which may imply that the affinities among the germplasm resources of *I. asprella* may also be closely related to social factors, such as human activities (Wu et al., 2018).

## 5 Conclusions

This study employed the identification and screening of polymorphic SSR molecular markers to investigate and analyze the genetic diversity and genetic structure of the *I. asprella* population. The results revealed substantial genetic differentiation and a high level of diversity within the *I. asprella* population. Significant genetic mobility and gene flow were observed among different *I. asprella* populations. This study provides valuable insights for future breeding programs aimed at developing new varieties of *I. asprella*.

## Data Availability

The original contributions presented in the study are included in the article/**Supplementary Material**. Further inquiries can be directed to the corresponding author.

## Supplementary material

Supplementary Figure 1 Electrophoretic detection map of PCR products from partial samples of *I. asprella* germplasm.
