# Supplementary material for: Genome-wide identification and development of SSR molecular markers for genetic diversity studies in Ilex asprella
Source: Front Plant Sci. 2025 May 23;16:1582154. doi: 10.3389/fpls.2025.1582154 (PMC12143170; doi:10.3389/fpls.2025.1582154)
Supplement: Supplementary Figure 1 — Electrophoretic detection map of PCR products from partial samples of I. asprella germplasm. [file Table1.docx]

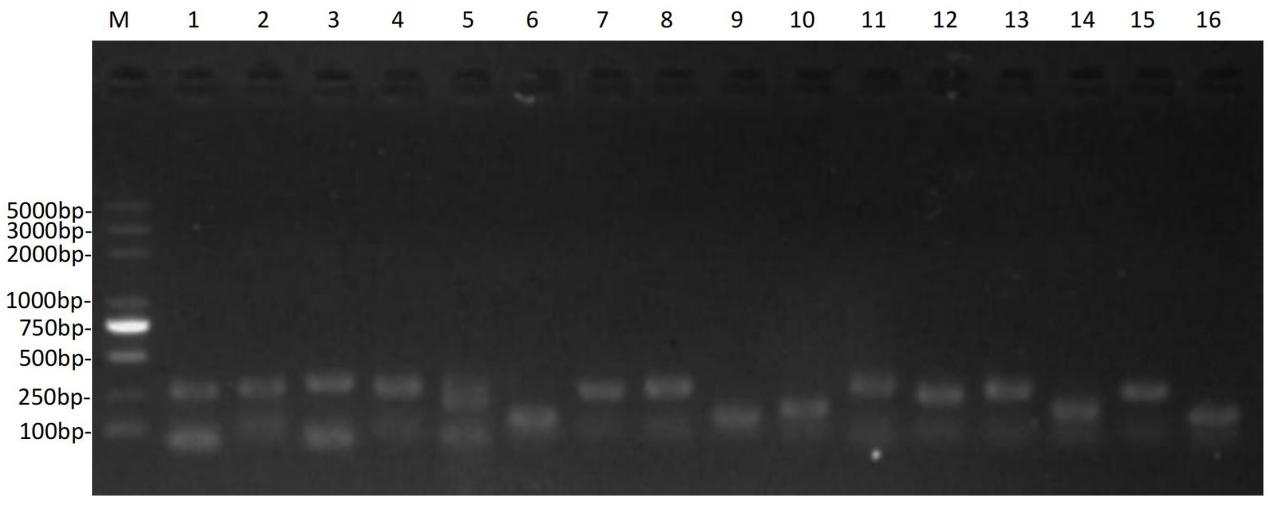
Figure S1 Electrophoretic detection map of PCR products from partial samples of *I. asprella* germplasm. Each lane represents the PCR product electrophoresis image of each germplasm DNA sample. M: DNA marker.
